# β-D-glucan inhibits endocrine-resistant breast cancer cell proliferation and alters gene expression

**DOI:** 10.3892/ijo.2014.2294

**Published:** 2014-02-10

**Authors:** ZAINAB M.T. JAFAAR, LACEY M. LITCHFIELD, MARGARITA M. IVANOVA, BRANDIE N. RADDE, NUMAN AL-RAYYAN, CAROLYN M. KLINGE

**Affiliations:** 1 Center of Biotechnology, Agricultural Research Directorate, Ministry of Science and Technology, Baghdad, Iraq;; 2 Department of Biochemistry and Molecular Biology, Center for Genetics and Molecular Medicine, University of Louisville School of Medicine, Louisville, KY 40292, USA

**Keywords:** endocrine-resistance, estrogen receptor, estradiol, tamoxifen, PCR array, transcription

## Abstract

Endocrine therapies have been successfully used for breast cancer patients with estrogen receptor α (ERα) positive tumors, but ∼40% of patients relapse due to endocrine resistance. β-glucans are components of plant cell walls that have immunomodulatory and anticancer activity. The objective of this study was to examine the activity of β-D-glucan, purified from barley, in endocrine-sensitive MCF-7 versus endocrine-resistant LCC9 and LY2 breast cancer cells. β-D-glucan dissolved in DMSO but not water inhibited MCF-7 cell proliferation in a concentration-dependent manner as measured by BrdU incorporation with an IC_50_ of ∼164±12 *μ*g/ml. β-D-glucan dissolved in DMSO inhibited tamoxifen/endocrine-resistant LCC9 and LY2 cell proliferation with IC_50_ values of 4.6±0.3 and 24.2±1.4 *μ*g/ml, respectively. MCF-10A normal breast epithelial cells showed a higher IC_50_ ∼464 *μ*g/ml and the proliferation of MDA-MB-231 triple negative breast cancer cells was not inhibited by β-D-glucan. Concentration-dependent increases in the BAX/BCL2 ratio and cell death with β-D-glucan were observed in MCF-7 and LCC9 cells. PCR array analysis revealed changes in gene expression in response to 24-h treatment with 10 or 50 *μ*g/ml β-D-glucan that were different between MCF-7 and LCC9 cells as well as differences in basal gene expression between the two cell lines. Select results were confirmed by quantitative real-time PCR demonstrating that β-D-glucan increased *RASSF1* expression in MCF-7 cells and *IGFBP3*, *CTNNB1* and ERβ transcript expression in LCC9 cells. Our data indicate that β-D-glucan regulates breast cancer-relevant gene expression and may be useful for inhibiting endocrine-resistant breast cancer cell proliferation.

## Introduction

Acquired resistance to antiestrogen or aromatase inhibitor therapy affects ∼40–50% of patients whose breast tumors are estrogen receptor α (ERα)-positive (1). Multiple mechanisms contribute to endocrine resistance and new therapies are needed to prevent endocrine resistance and treat these patients (2).

(1–3)β-D-glucans are diverse polysaccharides derived from plant cell walls composed of D-glucose monomers linked by (1–3)β-glycosidic bonds. The activities of β-glucans have been studied *in vivo* and *in vitro* (3). When ingested in plant materials, β-glucans are absorbed in the small intestine and taken up by macrophages. β-glucans are considered to be ‘biological response modifiers’ since they exhibit immunomodulatory, wound-healing, antiviral, antibacterial, anti-coagulatory and antitumoral activities (4). Because of their size, β-glucans work by binding to cell surface receptors (5). β-glucans act on several immune receptors, e.g., Dectin-1, complement receptor (CR3), scavenger receptors (SR), lactosylceramide (LacCer), and toll-like receptors, e.g., TLR-2/6, and trigger responses in macrophages, neutrophils, monocytes, natural killer cells, and dendritic cells *in vitro* (5,6). β-glucans themselves had no direct cytotoxic effects on a panel of common cancer cell lines including carcinoma, sarcoma and blastoma cells (6).

Cell inhibitory activities of β-glucans in cancer cells *in vitro* have also been reported. A water-soluble β-glucan extract from the mycelia of *Poria cocos* was reported to inhibit the viability (MTT assay) of MCF-7 breast cancer cells with an IC_50_ of 400 *μ*g/ml and to decrease cyclin D1 and cyclin E protein expression (7). The goal of this study was to examine the effect of a purified preparation of (1–3)β-D-glucan on the growth of endocrine-sensitive, estrogen receptor α (ERα)^+^ MCF-7 cells compared to normal breast epithelial (ERα^−^) MCF-10A cells; estradiol (E_2_)-independent, tamoxifen (TAM) and fulvestrant-resistant, ERα^+^ LCC9 (8,9) and LY2 (10,11) cells; and ‘triple negative/basal-like’ MDA-MB-231 (12) cells. Additionally, we examined the effect of β-D-glucan on the expression of a set of genes implicated in breast cancer in MCF-7 and LCC9 cells using a PCR array. While not affecting MCF-10A normal breast epithelial cell proliferation, our results indicate that β-D-glucan inhibits breast cancer cell proliferation and modulates gene expression independent of ERα activity and may be useful for inhibiting endocrine-resistant breast cancer cell proliferation.

## Materials and methods

### Cells

MCF-7 and MDA-MB-231 human breast cancer cells were purchased from ATCC (Manassas, VA, USA) and maintained in IMEM supplemented with 5% fetal bovine serum (Atlanta Biologicals, Lawrenceville, GA, USA) and 1% penicillin/streptomycin (Mediatech, Manassas, VA, USA) (13). LCC9 (8) and LY2 (10) cell lines were derived from MCF-7 cells by cultivation with the anti-estrogens ICI 182,780 (Fulvestrant) and LY 117018 respectively, and were graciously provided as a gift by Dr Robert Clarke, Georgetown University. MCF-10A cells are immortalized normal human breast epithelial cells that were also purchased from ATCC and grown in DMEM/F12 supplemented with 5% horse serum, 20 ng/ml epidermal growth factor (EGF), 16.67 *μ*g/ml insulin and 0.1% hydrocortisone (Sigma-Aldrich, St. Louis, MO, USA). Prior to treatment, the medium was replaced with phenol red-free IMEM supplemented with 5% dextran-coated charcoal-stripped FBS (DCC-FBS) and 1% penicillin/streptomycin for 48 h (referred to as ‘serum-starving’).

### Chemicals

Estradiol (E_2_) and 4-hydroxytamoxifen (4-OHT) were purchased from Sigma-Aldrich. ICI 182,780 was from Tocris (Ellisville, MO, USA). β-D-glucan was purchased from Sigma (cat. no. G6513, purity ∼97%). β-D-glucan was dissolved either in water or in DMSO (Sigma) by heating in a waterbath at 90°C for 4–5 min. Once dissolved, the β-D-glucan stocks were stored at −20°C until use. For all experiments, any effect(s) of DMSO were corrected in the calculations.

### Cell proliferation and cell death assays

The cells were seeded at a density of 5,000 cells/well in 96-well plates and were incubated for 24 h in growth medium prior to treatment. To initiate the experiment, the medium was removed and cells were treated with different concentrations of β-D-glucan (1–400 *μ*g/ml, as indicated in the Figures) and incubated for 72 h. Medium and treatments were changed after the first 48 h of incubation. For certain experiments, the cells were also treated with 100 nM or 1 *μ*M 4-OHT and β-D-glucan (10, 50 and 100 *μ*g/ml) to examine the potential synergistic effect of β-D-glucan with 4-OHT. Cell proliferation was determined after 72 h by measuring BrdU incorporation using an ELISA kit from Roche Applied Science (cat. 11647229001, Indianapolis, IN, USA) according to the manufacturer’s instructions. IC_50_ values were calculated using Excel.

Cell death was examined using the Live/Dead Viability/Cytotoxicity assay (Invitrogen), which determines intracellular esterase activity and plasma membrane integrity. In brief, 3×10^5^ cells were incubated with DMSO or increasing concentrations of β-D-glucan for 72 h. Cells were stained with the live and dead reagent [2 *μ*mol/l ethidium homodimers-1 (Eth-1) and 1 *μ*mol/l calcein-AM] and incubated at room temperature for 30 min. Fluorescence was read at 530 and 645 nM. The Live/Dead cell assay controls and calculations for % dead cells followed the manufacturer’s protocol.

### PCR arrays

MCF-7 or LCC9 cells were serum-starved, as above, for 48 h and then treated with DMSO (vehicle control), 10 or 50 *μ*g/ml β-D-glucan, 10 nM E_2_, or 100 nM 4-OHT. Total RNA was extracted using RNeasy Mini kit (Qiagen, Valencia, CA, USA). RNA quality was examined by NanoDrop Spectroscopy and cDNA synthesis was performed using the RT2 PCR Array First Strand kit (SABiosciences, Qiagen). RT2 Profiler PCR Array Breast Cancer SABiosciences cat no. PAHS-131ZA-12 contains 84 genes commonly involved in the dysregulation of signal transduction and other biological processes during breast carcinogenesis and in breast cancer cell lines plus 5 housekeeping genes http://www.sabiosciences.com/rt_pcr_product/HTML/PAHS-131Z.html. Breast cancer PCR arrays were performed according to the manufacturer’s instructions. Data analysis was performed using the web-based analysis tool (www.sabiosciences.com/pcrarraydataanalysis.php), including fold change and cluster analyses.

### Quantitative real-time PCR (qRT-PCR) analysis of mRNA expression

Total RNA was isolated from MCF-7 or LCC9 cells after 24-h treatment with DMSO (vehicle control), 10 nM E_2_, 100 nM 4-OHT, or 10 or 50 *μ*g/*μ*l β-D-glucan with RNeasy Mini kit (Qiagen) according to the manufacturer’s instructions. The quality and quantity of the isolated RNA was analyzed using NanoDrop spectroscopy. RNA (1 *μ*g) was reverse-transcribed using the High Capacity cDNA Reverse Transcription kit (Applied Biosystems, Carlsbad, CA, USA) and quantitation was performed using TaqMan primers and probes sets with TaqMan Gene Expression Master Mix (Applied Biosystems) and 18S was used for normalization. qRT-PCR was run using a ViiA7 Real-time PCR system (Applied Biosystems) with each reaction run in triplicate. Analysis and fold change were determined using the comparative threshold cycle (Ct) method. The change mRNA expression was calculated as fold-change, i.e., relative to DMSO-treated cells (control).

### Western blot analysis

Whole cell lysates were prepared from MCF-7 and LCC9 cells grown in phenol red-free IMEM + 5% DCC-stripped FBS for 48 h prior to addition of DMSO (vehicle control) or 10 or 50 *μ*g/ml β-D-glucan dissolved in DMSO for 24 h. Whole cell extracts (30 *μ*g protein) were separated on 10% SDS-PAGE gels and the resulting western blot was probed with HC-20 ERα antibody (Santa Cruz Biotechnology, Santa Cruz, CA, USA). The PVDF membrane was stripped and re-probed for β-actin (Sigma) for normalization. Chemiluminescent bands were visualized on a Carestream Imager using Carestream Molecular Imaging software (New Haven, CT, USA).

### Statistical analysis

Values are reported as ± SEM. Student’s t-test was used for comparisons between control and treatment. One-way ANOVA was used for multiple comparisons followed by Student-Newman-Keuls or Dunnett’s *post hoc* tests using GraphPad Prism. Values with p<0.05 were considered statistically significant.

## Results

### β-D-glucan dissolved in DMSO but not water inhibits MCF-7 cell proliferation

**Figure 1.**
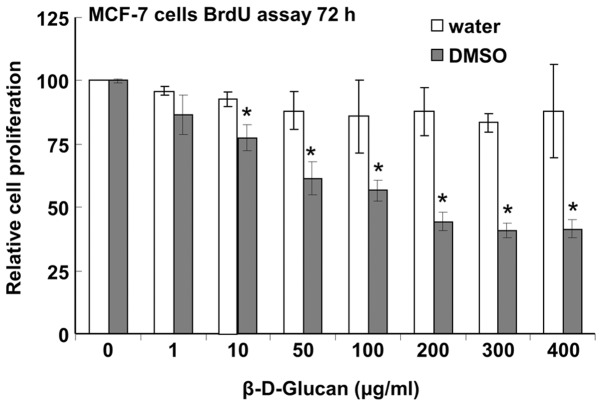
β-D-glucan dissolved in DMSO but not water inhibits MCF-7 cell proliferation. MCF-7 cells were incubated in phenol red-free IMEM + 5% DCC and the indicated concentrations of β-D-glucan dissolved in water or DMSO for a total of 72 h with a medium/treatment change after 48 h. Values are the mean ± SEM for 4 separate values in one experiment for β-D-glucan in water and 6 separate experiments (biological replicates) for β-D-glucan in DMSO. Values of β-D-glucan in DMSO were corrected for the inhibitory effect of DMSO on cell proliferation. ^*^p<0.05 vs. control (Student’s t-test). The IC_50_ value for β-D-glucan in MCF-7 cells was 164 *μ*g/ml (calculated in Excel).

Batch-to-batch variability of extracts of β-glucans leads to problematic heterogeneity of effects and controversy regarding their significance as potential anticancer agents (14). To obviate this issue, we purchased β-D-glucan purified from barley from Sigma and tested its activity in breast cancer cells. There was no inhibition of MCF-7 cell proliferation when cells were treated with β-glucan dissolved in boiling water, but cells were inhibited with an IC_50_ of ∼164±12 *μ*g/ml with β-glucan dissolved in DMSO (Fig. 1).

### β-D-glucan inhibits MCF-10A, but not HEK-293 cell proliferation

**Figure 2.**
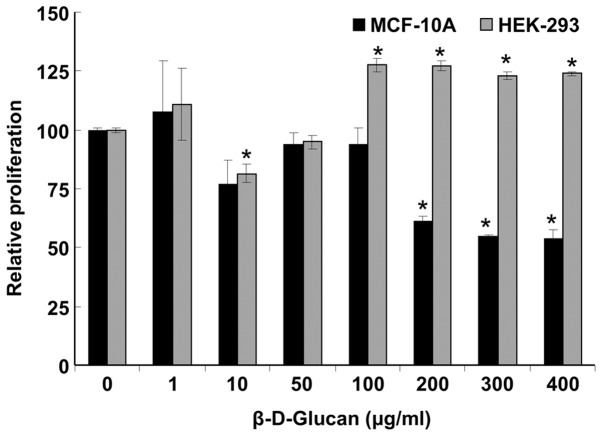
β-D-glucan inhibits MCF-10A but not HEK-293 cell proliferation. MCF-10A and HEK-293 cells were incubated in phenol red-free IMEM + 5% DCC and the indicated concentrations of β-D-glucan dissolved in DMSO for a total of 72 h with a medium/treatment change after 48 h. Values are the BrdU incorporation absorbances normalized to DMSO (zero) and are the mean ± SEM for 4 separate values in one experiment. Values of β-D-glucan in DMSO were corrected for the inhibitory effect of DMSO on cell proliferation. ^*^p<0.05 vs. control (Student’s t-test). The IC_50_ value for β-D-glucan in MCF-10A cells was 464 *μ*g/ml (calculated in Excel).

Next, we examined if DMSO-solubilized β-D-glucan affected the growth of ‘normal’ cells using MCF-10A immortalized breast epithelial cells and HEK-293 cells (Fig. 2). β-D-glucan inhibited MCF-10A proliferation with an IC_50_ of ∼464 *μ*g/ml, but had no significant inhibitory effect on HEK-293 cells, although a somewhat ‘U-shaped’ response was detected, i.e., apparent inhibition at 10 *μ*g/ml and stimulation at 100–400 *μ*g/ml.

### β-D-glucan inhibits the proliferation of endocrine-resistant cells

**Figure 3.**
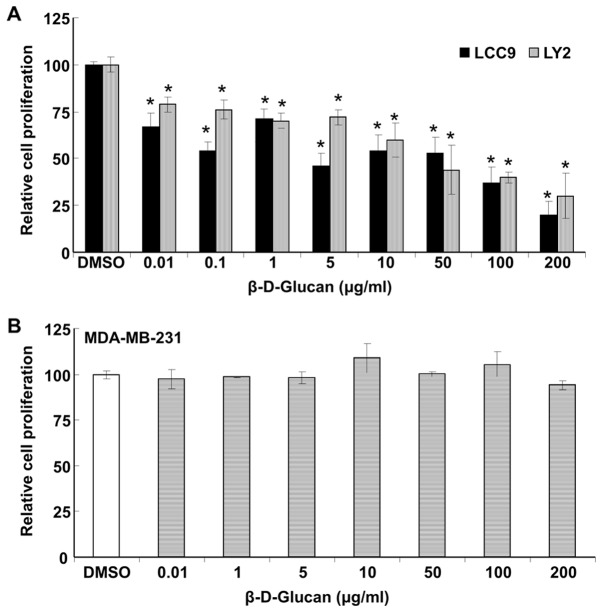
β-D-glucan inhibits the proliferation of endocrine-resistant breast cancer cells. LCC9 and LY2 endocrine-resistant breast cancer cells (A) and MDA-MB-231 triple negative breast cancer cells (B) were incubated in phenol red-free IMEM + 5% DCC and the indicated concentrations of β-D-glucan dissolved in DMSO for a total of 72 h with a medium/treatment change after 48 h. Values are the BrdU incorporation absorbances normalized to DMSO (zero) and are the mean ± SEM for 3 separate experiments. Values of β-D-glucan in DMSO were corrected for the inhibitory effect of DMSO on cell proliferation. ^*^p<0.05 vs. control (Student’s t-test).

The development of acquired resistance to tamoxifen and other endocrine agents is a major concern in breast cancer patients. We examined if DMSO-solubilized β-D-glucan inhibited the growth of LCC9 and LY2 endocrine-resistant breast cancer cells (Fig. 3A). β-D-glucan inhibited the proliferation of each cell line, with IC_50_ values of 4.6±0.3 and 24.2±1.4 *μ*g/ml for LCC9 and LY2, respectively. In contrast, β-D-glucan had no effect on MDA-MB-231 triple-negative/basal-like breast cancer cells (Fig. 3B).

**Figure 4.**
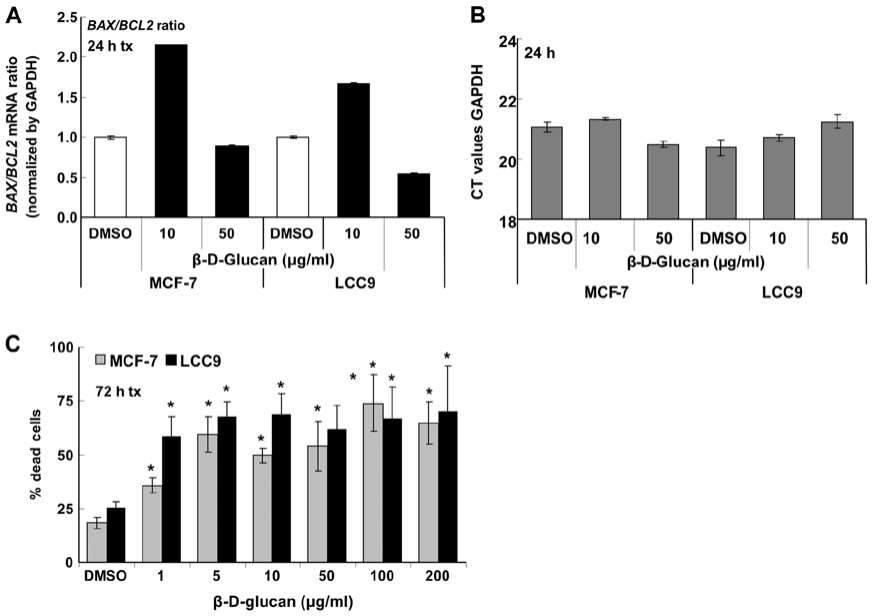
β-D-glucan increases apoptosis and cell death in MCF-7 and LCC9 cells. (A) MCF-7 tamoxifen-sensitive and LCC9 tamoxifen-resistant breast cancer cells were incubated in phenol red-free IMEM + 5% DCC for 48 h prior to addition of the indicated concentrations of β-D-glucan dissolved in DMSO or DMSO as vehicle control for 24 h. *BAX* and *BCL2* mRNA transcript expression was normalized by *GAPDH* (B) and the fold relative to DMSO (vehicle control) was set to one. (B) qPCR for *GAPDH* expression is given as CT values. For (A) and (B), the values are the average ± SEM of triplicate determinations within one experiment. (C) MCF-7 and LCC9 cells were incubated in phenol red-free IMEM + 5% DCC and the indicated concentrations of β-D-glucan dissolved in DMSO or DMSO as vehicle control for 72 h with a medium/treatment change after 48 h. Live/Dead Viability/Cytotoxicity assay was performed as described in Materials and methods. Values are the % of dead cells measured by uptake of ethidium homodimer-1 and fluorescence emission at 645 nm. Values are the average of 4 replicates within one experiment. ^*^p<0.05 vs. control (Student’s t-test).

To examine the possible contribution of apoptosis to the observed decrease in MCF-7 and LCC9 cell viability with β-D-glucan treatment, we measured the expression of *BAX* (pro-apoptotic) and *BCL2* (anti-apoptotic) in MCF-7 and LCC9 cells treated with vehicle (DMSO), 10 or 50 *μ*g/ml β-D-glucan (Fig. 4A). *GAPDH* mRNA transcript levels were not affected by β-D-glucan (Fig. 4B). An increased *BAX/BCL2* is an indicator of apoptosis (15). As reported previously (16), basal *BCL2* expression was higher in the endocrine-resistant LCC9 cells compared to parental, endocrine-sensitive MCF-7 cells (data not shown). β-D-glucan (10 *μ*g/ml) increased the *BAX/BCL2* ratio in both cell lines, but that increase was not sustained at 50 *μ*g/ml β-D-glucan.

Live/Dead cell assays were performed to examine cell death through determination of intracellular esterase activity and plasma membrane integrity (Fig. 4C). The data show that β-D-glucan increases cell death in both MCF-7 and LCC9 cells with more death in LCC9 versus MCF-7 cells at 1 *μ*g/ml β-D-glucan. There appears to be a saturation, with maximal ∼70% cell death in both cell lines.

### β-D-glucan has no effect on TAM-sensitivity of MCF-7 or LCC9 cells

**Figure 5.**
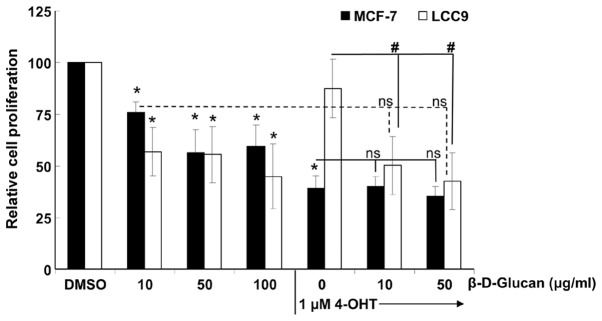
β-D-glucan does not synergize with 4-hydroxytamoxifen to inhibit cell proliferation. MCF-7 tamoxifen-sensitive and LY2 tamoxifen-resistant breast cancer cells were incubated in phenol red-free IMEM + 5% DCC and the indicated concentrations of β-D-glucan dissolved in DMSO, 1 *μ*M 4-OHT, or the combination of 1 *μ*M 4-OHT + 10 or 50 *μ*g/ml β-D-glucan, as indicated, for a total of 72 h with a medium/treatment change after 48 h. Values are the BrdU incorporation absorbances normalized to DMSO (zero) and are the mean ± SEM for 3 separate experiments. Values of β-D-glucan in DMSO were corrected for the inhibitory effect of DMSO on cell proliferation. ^*^p<0.05 vs. control. ^#^p<0.05 vs. 1 *μ*M 4-OHT alone (one-way ANOVA followed by Dunnett’s *post hoc* test). ns, not statistically different from the same treatment in that cell line, i.e., dotted line indicates that the values for LCC9 with 10 or 50 *μ*g/ml β-D-glucan are not different from the values for LCC9 with 4-OHT + 10 or 50 *μ*g/ml β-D-glucan.

A β-D-glucan extract called schizophyllan that was extracted from the mushroom *Schizophyllum commune* by boiling in water showed no additive effect with TAM treatment in suppressing PCNA staining in DMBA-induced mouse mammary tumors, but inhibited TAM-induced PCNA staining in liver tumors of the same mice (17). We tested if β-D-glucan synergized with 4-OHT to inhibit MCF-7 endocrine-sensitive and LCC9 endocrine-resistant cell growth. There was no effect of β-D-glucan on the inhibition of MCF-7 cell growth by 4-OHT, nor was there any effect of 4-OHT on the inhibition of LCC9 cell proliferation by β-D-glucan (Fig. 5).

### β-D-glucan inhibits NRF-1 expression in MCF-7 cells

**Figure 6.**
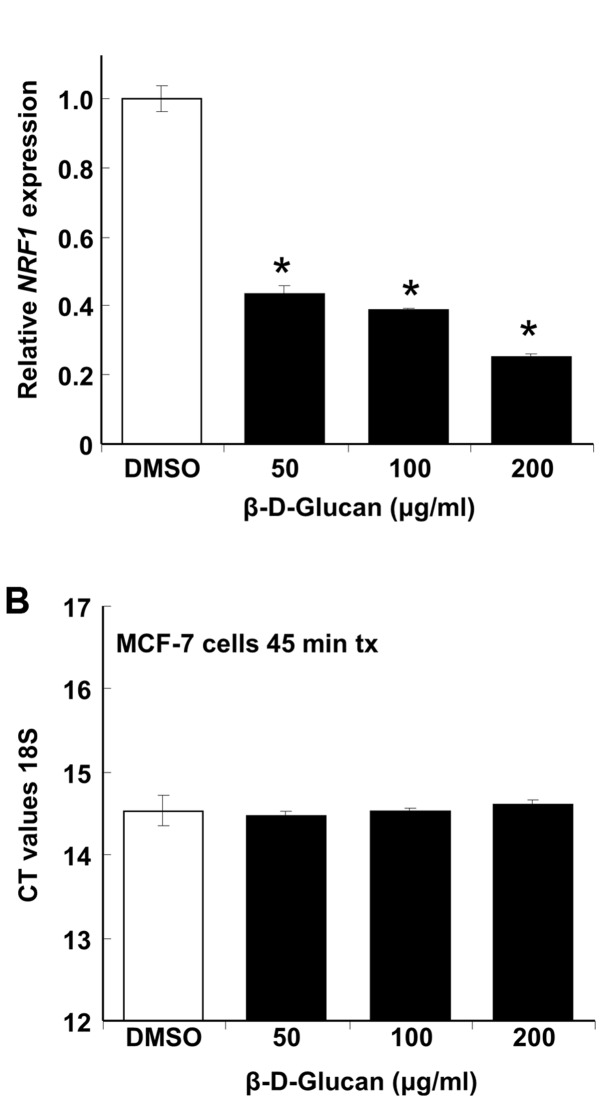
β-D-glucan rapidly inhibits *NRF1* expression in MCF-7 cells. MCF-7 cells were grown in phenol red-free IMEM + 5% DCC for 48 h prior to addition of the indicated concentrations of DMSO-dissolved β-D-glucan for 45 min. (A) qPCR for NRF1 mRNA expression was normalized to 18S rRNA. ^*^p<0.05 vs. control (Student’s t-test). (B) qPCR for 18S expression is given as CT values.

Nuclear respiratory factor-1 (NRF-1) is a master transcription factor regulating the transcription of nuclear genes controlling many aspects of mitochondrial function including respiration (18). Knockdown of NRF-1 in MCF-7 breast cancer cells using siRNA increases apoptosis and overexpression of NRF-1 inhibits 4-OHT-mediated apoptosis (19). We tested the hypothesis that the inhibition of MCF-7 cell proliferation and viability by β-D-glucan (Figs. 1 and 4) would be reflected in inhibition of NRF-1 expression. β-D-glucan rapidly inhibited NRF-1 transcription in a concentration-dependent manner without affecting 18S rRNA expression (Fig. 6). The rapid inhibition of NRF-1 transcription is commensurate with plasma membrane effects of β-D-glucan.

### β-D-glucan affects breast cancer gene expression in a cell type-dependent manner

To identify other potential breast cancer-associated genes regulated by β-D-glucan, we performed PCR array analysis on 84 genes commonly dysregulated during breast carcinogenesis and in breast cancer cell lines (Breast Cancer PCR array PAHS-131Z, SABiosciences). For these experiments, MCF-7 or LCC9 cells were serum-starved for 48 h in phenol red-free medium and then treated in duplicate with DMSO (vehicle control), 10 nM E_2_, 100 nM 4-OHT, or 10 or 50 *μ*g/ml β-D-glucan for 24 h. Using a 2-fold cut off, β-D-glucan altered the expression of 17 genes in MCF-7 cells: 8 downregulated and 9 upregulated. Some, but not all, genes showed a dose-dependent effect of β-D-glucan, e.g., *IGFBP3* showed a greater decrease with 50 than 10 *μ*g/ml β-D-glucan (Table I). In the group of genes with increased expression in MCF-7 with β-D-glucan, some β-D-glucan regulated genes had a similar expression pattern as with E_2_ treatment and others showed similarity with 4-OHT treatment (Table II). β-D-glucan altered the expression of 8 genes in LCC9: 3 downregulated and 5 upregulated (Tables III and IV). E_2_ altered the expression of 17 genes in MCF-7: 9 downregulated and 8 upregulated. 4-OHT altered the expression of 8 genes in MCF-7: 1 downregulated and 7 upregulated. E_2_ altered the expression of 5 genes in LCC9: 2 downregulated and 3 upregulated. 4-OHT altered the expression of 10 genes in LCC9: 5 downregulated and 5 upregulated. We note that 17 genes showed lower expression in LCC9 than MCF-7 cells (Table V). Conversely, 31 genes showed higher expression in LCC9 than MCF-7 cells (Table VI).

### Confirmation of select changes in breast cancer gene expression by qRT-PCR

To determine if the changes detected in the PCR array after treatment of MCF-7 and LCC9 cells with β-D-glucan were reproducible by qRT-PCR, five gene targets were selected for verification: *RASSF1, CTNNB1, IGFBP3, ESR2* (ERβ), and *AR* (Tables I–III, V and VI). 18S was used for normalization and was not significantly different between the two cell lines or with β-D-glucan treatment (data not shown). The rationale for selecting these genes for follow-up is based on their regulation by β-D-glucan in the PCR array: *RASSF1* was increased in MCF-7 (E_2_ and 4-OHT also increased *RASSF1*, Table II); *CTNNB1* was decreased in MCF-7 and LCC9; *IGFBP*3 and *ESR2* were increased in LCC9; *AR* was decreased in MCF-7. Further rationale is based on their roles in breast cancer. *RASSF1A* is a tumor suppressor gene that is downregulated by hypermethylation in various human cancers including breast cancer (20,21). *CTNNB1* encodes β-catenin, an adherens junction protein that plays a critical role in cellular adhesion and intercellular communication which also translocates to the nucleus to activate genes whose promoters contain binding sites for Tcf/Lef (22). Activation of the Wnt/β-catenin pathway plays a role in breast tumorigenesis (23,24). Insulin-like growth factor (IGF) binding protein 3 (IGFBP3) is a major carrier of IGF1 and IGF2 in circulation and IGFBP3 levels are reduced in breast cancer patients, giving rise to higher free IGF1 levels and poor prognosis (25,26). IGFBP3 also stimulates or inhibits normal and neoplastic breast cell proliferation by stimulating EGFR activation or stimulating apoptotic effector proteins (27,28). E_2_ stimulates IGFBP3 expression in MCF-7 cells (29) and both E_2_ and 4-OHT increased *IGFBP* transcript expression in MDA-MB-231 triple negative breast cancer cells transfected with ERα (30). When IGFBP3 was transfected into LCC9 endocrine-resistant breast cancer cells, it was shown, by co-immunoprecipitation, to interact with the 78-kDa glucose regulated protein (GRP78), which is highly expressed in LCC9 and other endocrine-resistant breast cancer cells (31), and to dissociate caspase 7 from GRP78, thus sensitizing LCC9 cells to growth inhibition by fulvestrant (ICI 182,780) (32). Increased AR expression is found in tamoxifen-resistant breast tumors and overexpression of AR in MCF-7 cells caused the cells to become resistant to growth inhibition by tamoxifen (33).

**Figure 7.**
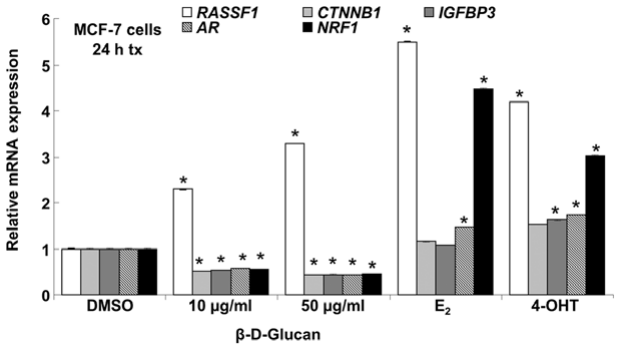
Quantitative real-time PCR analysis of select targets regulated by β-D-glucan in MCF-7 cells. Cells were grown in phenol red-free IMEM + 5% DCC for 48 h prior to addition of DMSO (vehicle control), 10 nM E_2_, 100 nM 4-OHT or the indicated concentrations of DMSO-dissolved β-D-glucan for 24 h. qPCR for each target gene was normalized to 18S rRNA and values were compared to fold expression in vehicle (DMSO)-treated MCF-7 cells. Values are the average of triplicate determinations ± SEM within one experiment.

The increase in *RASSF1* transcript expression (Table II) was reproducibly increased by treatment with β-D-glucan, E_2_ and 4-OHT in MCF-7 cells (Fig. 7). The inhibition of *CTNNB1, IGFBP3* and *AR* (Table I) by β-D-glucan in MCF-7 cells was confirmed; however, E_2_ and 4-OHT did not significantly inhibit the expression of these genes (Fig. 7), a result different from that detected in the PCR array. Since qRT-PCR is the accepted standard to compare transcript levels, these data suggest that E_2_ and 4-OHT may not significantly inhibit *CTNNB1, IGFBP3* and *AR* in MCF-7 cells with 24 h of treatment. In fact, *CTNNB1* (β-catenin) transcript expression was statistically increased by 4-OHT in MCF-7, although only by 0.5-fold (Fig. 7).

**Figure 8.**
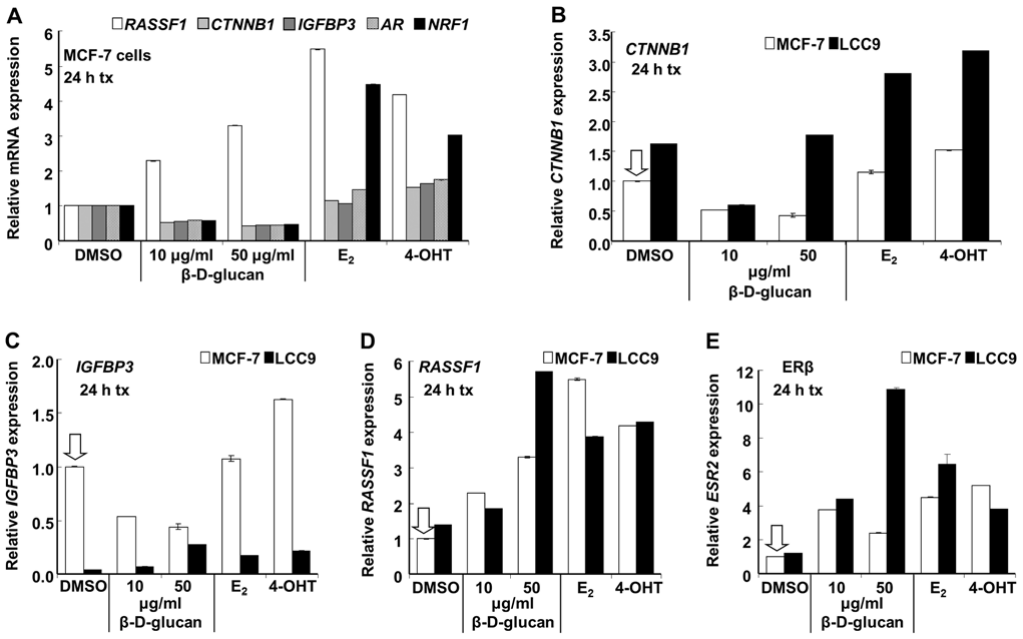
Quantitative real-time PCR analysis of select targets regulated by β-D-glucan in MCF-7 and LCC9 cells. Cells were grown in phenol red-free IMEM + 5% DCC for 48 h prior to addition of DMSO (vehicle), 10 nM E_2_, 100 nM 4-OHT or the indicated concentrations of DMSO-dissolved β-D-glucan for 24 h. qPCR for each target gene was normalized to 18S and values were compared to fold expression in vehicle (DMSO)-treated MCF-7 cells. Values are the average of triplicate determinations ± SEM within one experiment. (A) *RASSF1, CTNNB1, IGFBP3, AR* and *NRF1* transcript expression in MCF-7 cells relative to DMSO control. (B) *CTNNB1*, (C) *IGFBP3*, (D) *RASSF1* and (E) *ESR2* (ERβ) transcript expression in MCF-7 and LCC9 cells relative to DMSO control.

*CTNNB1* (β-catenin) expression was decreased by β-D-glucan in a concentration-dependent manner in LCC9 in the PCR array (Table III) and by 10 *μ*g/ml β-D-glucan as assessed by qRT-PCR (Fig. 7). However, 50 *μ*g/ml β-D-glucan, E_2_ and 4-OHT increased *CTNNB1* in LCC9 cells. *CTNNB1* basal expression was ∼63% higher in LCC9 than MCF-7 (Fig. 8A), although this was not detected in the PCR array (Table VI). β-catenin mRNA and protein expression is increased in another tamoxifen-resistant cell line derived from MCF-7 cells (34) and in breast tumors (35). The increase in *CTNNB1* transcript expression with E_2_ and 4-OHT was significant in both MCF-7 and LCC9 cells, although the fold-response, 1.7- and 2-fold respectively, compared to basal (DMSO), was higher in LCC9 cells. This increase in β-catenin expression would be expected to interact with and increase TCF/LEF1 target gene expression in these cells, a pathway contributing to breast cancer progression (23).

AR (androgen receptor, AR, *NR3C4*) expression was reduced by β-D-glucan in MCF-7 cells while E_2_ and 4-OHT slightly increased AR expression.

We confirmed that β-D-glucan inhibited NRF-1 transcription in MCF-7 cells (Figs. 6 and 7) whereas E_2_ and 4-OHT increased NRF-1 expression, as previously reported (19,36). Basal NRF-1 transcript expression was higher in LCC9 cells and was increased by β-D-glucan and inhibited by E_2_ and 4-OHT (Fig. 8B).

Expression of *IGFBP3* (insulin-like growth factor binding protein 3) was 25.7-fold lower in LCC9 than MCF-7 (Table V). This result was confirmed by qRT-PCR (Fig. 8C). This is consistent with a previous report that IGFBP3 protein secretion was reduced in tamoxifen-resistant LY2 and ZR-75-9a1 cells (37). *IGFBP3* (which sequesters IGF) was increased by β-D-glucan and E_2_ in LCC9 cells (Table IV). These results were confirmed by qRT-PCR. 4-OHT also increased *IGFBP3* in LCC9 cells. In MCF-7 cells, β-D-glucan inhibited *IGFBP3* transcript expression whereas 4-OHT increased *IGFBP3* expression.

**Figure 9.**
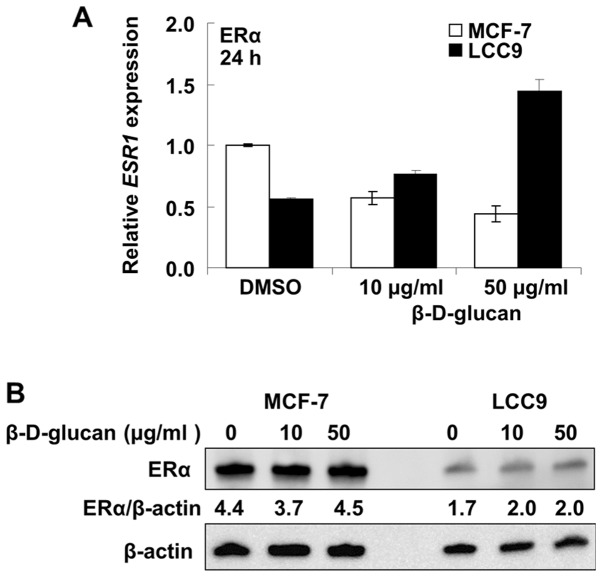
β-D-glucan affects ERα expression in MCF-7 and LCC9 cells. Cells were grown in phenol red-free IMEM + 5% DCC-stripped FBS for 48 h prior to addition of DMSO (vehicle control) or 10 or 50 *μ*g/ml β-D-glucan dissolved in DMSO for 24 h. (A) *ESR1* transcript levels were measured by qPCR relative to 18S and are the average of triplicate determinations ± SEM within one experiment. Values are relative to MCF-7 cells treated with DMSO showing that ERα mRNA expression is lower in LCC9 relative to MCF-7 cells. (B) Whole cell extracts (30 *μ*g protein) were separated on 10% SDS-PAGE gels and the resulting western blot was probed with ERα antibody and the full length 66 kDa ERα band is shown. The PVDF membrane was stripped and re-probed for β-actin for normalization. Values are the ERα/β-actin ratio.

*ESR2* (ERβ) and *RASSF1* (Ras-association domain family protein 1) showed higher expression in LCC9 than MCF-7 cells in the PCR array (Table VI). This may be surprising since ERβ inhibits the proliferative activity of ERα (38) and *RASFF1* is a tumor suppressor gene whose inactivation by hypermethylation of a CpG island in the gene promoter (39) has been implicated in a wide variety of sporadic human cancers, including breast cancer (20). Results were confirmed by qRT-PCR (Fig. 8D and E). As in MCF-7 cells, β-D-glucan, E_2_ and 4-OHT increased *RASSF1* expression in LCC9 cells (Fig. 8E). In contrast to the increase in ERβ, β-D-glucan reduced *ESR1* (ERα) mRNA transcript levels in MCF-7 cells and increased ERα mRNA expression in LCC9 cells (Fig. 9A). ERα protein expression was unaffected (change <10%) by β-D-glucan treatment in MCF-7 cells and increased ∼17% in LCC9 cells (Fig. 9B).

## Discussion

Multiple mechanisms contribute to acquired endocrine resistance in breast cancer and new therapies are needed to prevent disease recurrence (2). Here we report that DMSO-solubilized β-D-glucan inhibited the proliferation of endocrine-sensitive MCF-7 and endocrine-resistant LCC9 and LY2 breast cancer cell lines, but did not inhibit the proliferation of MDA-MB-231 TNBC cells. Notably the IC_50_ values for the breast cancer cell lines were significantly lower than that for MCF-10A immortalized breast epithelial cells. We also report that β-D-glucan increases cell death in both MCF-7 and LCC9 cells with more death in LCC9 versus MCF-7 cells at 1 *μ*g/ml β-D-glucan. We found that 10 *μ*g/ml β-D-glucan increased the *BAX/BCL2* ratio in both MCF-7 and LCC9 cells, but that increase was not sustained at 50 *μ*g/ml β-D-glucan. Given the decrease in NRF-1 transcription with β-D-glucan, it is possible that β-D-glucan is inhibiting mitochondrial function due to toxicity at the 50 *μ*g/ml β-D-glucan concentration. Further studies will be required to probe mechanisms of cell death in response to β-D-glucan. We had hoped that β-D-glucan would synergize with 4-OHT to inhibit breast cancer cell proliferation, but it did not. These findings agree with the lack of effect of β-D-glucan and TAM in DMBA-induced mammary tumors (17).

A previous study reported that water-soluble β-glucan extract from the mycelia of *Poria cocos* inhibited MCF-7 cell viability with an IC_50_ of 400 *μ*g/ml (7). Others reported no inhibition of cell viability, measured by MTT assay, using *Clitocybe alexandri* and *Lepista inversa* mushroom extracts dissolved in boiling water, but when dissolved in methanol or ethanol, MCF-7 cells were inhibited with an IC_50_ of 20–80 *μ*g/ml, depending on which solvent and which mushroom extract was tested (40). Our data are in agreement with a lack of inhibitory activity of β-glucan dissolved in water, and indicate that anti-proliferative activity in MCF-7 cells β-glucan depends on solubilization in an organic solvent. Future studies are needed to identify the active components of the DMSO-solubilized β-D-glucan.

HEK-293 cells showed a non-monotonic or possible ‘U-shaped’ dose response to β-D-glucan in which there was a slight inhibition at a low concentration (10 *μ*g/ml), but increasing concentrations resulted in stimulation of cell proliferation. U-shaped or other non-monotonic dose-responses, referred to as ‘hormesis’, have been reported in studies of a variety of chemotherapeutics, cytokines, rosiglitazone and other clinically used drugs (41), endocrine-disruptors (42), and phytoestrogens indicating that ‘compounds in a cellular context, can have opposite effects at different concentrations’ (43). Mechanistically, the mechanism for the lack of linear response in HEK-293 cells is unknown, but we may speculate that at lower β-D-glucan concentrations a higher affinity antiproliferative response is triggered whereas at higher concentrations, i.e., a lower affinity response, there is an increase in cell proliferation which appears to reach saturation.

A PCR array identified potential breast cancer-associated genes regulated by β-D-glucan and selected genes were verified by qRT-PCR. AR (NR3C4) expression was reduced by β-D-glucan in MCF-7 cells. There is one report that AR overexpression in MCF-7 cells reduced ERα and caused cells to become tamoxifen-resistant (33). Chromatin immunoprecipitation sequencing (ChIP-seq) and microarray expression profiling has revealed significant cross-talk in gene regulation between AR and ERα in ZR-75-1 human breast cancer cells (44). The role of AR suppression by β-D-glucan on the β-D-glucan inhibition of cell proliferation in MCF-7 cells is unknown and may be investigated in future studies.

β-D-glucan, E_2_ and 4-OHT increased *RASSF1* expression in MCF-7 and LCC9 cells. A reduction in *RASSF1* has been shown to correlate with tamoxifen resistance (45) and the ability of β-D-glucan to increase *RASSF1* expression may correspond to the observed inhibition of cell proliferation and increase in cell death. Further studies will be required to determine the downstream targets regulated by β-D-glucan-induced *RASSF1*. Likewise, the increase in ERβ mRNA in LCC9 cells treated with β-D-glucan is another logical follow-up for this study to further characterize the mechanisms by which β-D-glucan inhibits breast cancer cell proliferation *in vitro*.

## Figures and Tables

**Table I.** Genes with decreased expression following treatment with β-D-glucan, E_2_ and/or 4-OHT in MCF-7 endocrine-sensitive breast cancer cells.

Symbol	10 *μ*g/ml β-D-glucan	50 *μ*g/ml β-D-glucan	E_2_	4-OHT
*AR*	1.1	−1.2	**−2.1**	−1.2
*ATM*	−1.3	−1.6	**−4.3**	−1.9
*CCND2*	−1.4	1.0	**−2.1**	−1.0
*CDKN1C*	**−2.2**	**−2.4**	**−2.0**	−1.6
*CSF1*	−1.1	−1.2	**−2.4**	1.1
*CTNNB1*	−1.0	**−4.3**	−1.2	1.0
*ERBB2*	−1.6	−1.9	**−2.2**	1.4
*GRB7*	−1.6	−1.7	**−2.4**	1.0
*IGFBP3*	−1.7	**−2.2**	−1.7	1.2
*MUC1*	**−2.1**	−1.4	−1.4	−1.0
*NOTCH1*	−1.6	−1.0	**−2.3**	−1.5
*PLAU*	**−2.3**	**−2.4**	−1.5	1.1
*RARB*	**−3.5**	**−2.2**	**−3.3**	**−2.3**
*SLIT2*	−1.5	**−2.7**	**−3.0**	1.5
*SNAI2*	**−2.4**	−1.5	−1.1	1.9

Values in bold are greater than the 2-fold cut off in the Breast Cancer PCR array (PAHS-131Z, SABiosciences).

**Table II.** Genes with increased expression following treatment with β-D-glucan, E_2_ and/or 4-OHT in MCF-7 endocrine-sensitive breast cancer cells.

Symbol	10 *μ*g/ml β-D-glucan	50 *μ*g/ml β-D-glucan	E_2_	4-OHT
*BIRC5*	**2.6**	**3.0**	**2.2**	1.0
*BRCA1*	**2.7**	**2.6**	**2.5**	−1.6
*BRCA2*	**2.3**	**2.7**	**2.5**	−1.3
*CCNA1*	**2.1**	**2.6**	**5.3**	1.2
*CTSD*	**2.2**	1.7	1.8	1.0
*EGF*	1.2	−1.2	1.3	**2.5**
*GLI1*	1.7	1.2	−1.6	**2.3**
*GSTP1*	1.8	1.1	−1.2	**3.5**
*KRT5*	1.7	−1.0	−1.2	**2.2**
*MKI67*	1.9	**2.6**	**2.2**	1.1
*NME1*	1.7	1.8	**2.1**	−1.2
*PGR*	**3.8**	**2.9**	**2.9**	−1.2
*PTGS2*	**2.5**	1.8	1.9	1.1
*RASSF1*	**4.0**	**3.8**	**2.2**	**3.2**
*SERPINE1*	1.3	1.4	**2.0**	**2.4**
*TP73*	1.2	−1.2	−1.3	**6.5**

Values in bold are greater than the 2-fold cut off in the Breast Cancer PCR array (PAHS-131Z, SABiosciences).

**Table III.** Genes with decreased expression following treatment with β-D-glucan, E_2_ and/or 4-OHT in LCC9 endocrine-resistant breast cancer cells.

Symbol	10 *μ*g/ml β-D-glucan	50 *μ*g/ml β-D-glucan	E_2_	4-OHT
*ADAM23*	1.2	−1.6	1.2	**−2.3**
*BRCA2*	−1.4	−1.6	**−2.3**	**−2.4**
*CDH13*	**−2.5**	−1.8	**−2.6**	−1.8
*CDKN1C*	**−2.4**	−1.4	−1.6	**−3.0**
*CTNNB1*	−1.4	**−2.1**	−1.7	**−2.1**
*ID1*	1.6	1.2	−1.7	**−2.4**

Values in bold are greater than the 2-fold cut off in the Breast Cancer PCR array (PAHS-131Z, SABiosciences).

**Table IV.** Genes with increased expression following treatment with β-D-glucan, E_2_ and/or 4-OHT in LCC9 endocrine-resistant breast cancer cells.

Symbol	10 *μ*g/ml β-D-glucan	50 *μ*g/ml β-D-glucan	E_2_	4-OHT
*EGF*	1.8	**5.1**	**4.6**	**2.1**
*GLI1*	**4.9**	**7.4**	**3.3**	**8.3**
*HIC1*	1.4	1.4	1.6	**2.5**
*IGF1*	**5.6**	1.4	1.7	**2.9**
*IGFBP3*	**2.0**	**2.0**	**2.9**	1.1
*PTGS2*	**2.2**	1.2	1.7	1.1
*TWIST1*	−1.2	−1.1	1.5	**3.7**

Values in bold are greater than the 2-fold cut off in the Breast Cancer PCR array (PAHS-131Z, SABiosciences).

**Table V.** Genes with lower expression in LCC9 endocrine-resistant vs. MCF-7 endocrine-sensitive breast cancer cells.

Symbol	Fold
*ABCG2*	−35.2
*BCL2*	−2.1
*CCND2*	−2.0
*CDKN1A*	−5.0
*EGF*	−2.6
*EGFR*	−9.1
*GATA3*	−4.1
*ID1*	−2.2
*IGF1R*	−2.5
*IGFBP3*	−25.7
*JUN*	−5.5
*KRT18*	−3.9
*KRT8*	−4.6
*MGMT*	−2.5
*PLAU*	−2.0
*SLC39A6*	−3.7
*THBS1*	−14.1

All genes included show >2-fold changes in the Breast Cancer PCR array (PAHS-131Z, SABiosciences).

**Table VI.** Genes with higher expression in LCC9 endocrine-resistant vs. MCF-7 endocrine-sensitive breast cancer cells.

Symbol	Fold
*ABCB1*	2.1
*ADAM23*	11.4
*BIRC5*	5.9
*BRCA1*	2.8
*BRCA2*	7.2
*CCNA1*	78.0
*CDH13*	4.3
*CDK2*	4.1
*CDKN1C*	2.3
*CDKN2A*	2.1
*CST6*	2.1
*ESR2*	2.1
*GLI1*	2.2
*GSTP1*	28.2
*HIC1*	4.0
*IGF1*	2.1
*IL6*	2.1
*KRT5*	2.1
*MAPK1*	4.4
*MMP2*	2.1
*MMP9*	3.0
*MYC*	3.6
*PGR*	4.5
*PRDM2*	2.2
*PTEN*	5.2
*RASSF1*	2.1
*SERPINE1*	85.4
*SFRP1*	2.1
*TWIST1*	5.1
*VEGFA*	2.6
*XBP1*	3.7

All genes included show >2-fold changes in the Breast Cancer PCR array (PAHS-131Z, SABiosciences).
